# Primary lymphoma of the tibia in children: Two case reports

**DOI:** 10.1097/MD.0000000000018807

**Published:** 2020-01-24

**Authors:** Haiqiang Suo, Li Fu, Zhiwei Wang, Hanguang Liang, Zhe Xu, Wei Feng

**Affiliations:** a Department of Bone and Joint, The First Hospital of Jilin; b Department of Obstetrics and Gynecology, The Second Hospital of Jilin University Changchun, Jilin, China.

**Keywords:** children, non-Hodgkin lymphomas, primary lymphoma of bone, tibia

## Abstract

**Rationale:**

Primary lymphoma of the bones (PLB) is a rare extranodal non-Hodgkin lymphoma (NHL) that is particularly rare in children. The clinical presentation and radiological features of PLB are often nonspecific, making clinical diagnosis challenging and misdiagnosis frequent. Here, we report 2 children with PLB focusing on clinical presentation, differential diagnosis, and treatment outcomes.

**Patients concerns:**

A 9-year-old boy presented with left knee swelling and pain for 4 months after a fall. He was previously misdiagnosed with traumatic soft tissue injury. The second patient was an 11-year-old boy with a 6-month history of intermittent left knee pain. He was previously misdiagnosed with bone tuberculosis and chronic osteomyelitis.

**Diagnoses:**

A 9-year-old boy showed an abnormal signal of the left tibia metaphysis, diaphysis, and epiphysis, and tibia with periosteal reactions and surrounding soft tissue swelling. Tumor biopsy and immunohistochemistry confirmed a diagnosis of B-cell lymphoblastic lymphoma.

An 11-year-old boy showed a permeative lesion in the metaphysis and diaphysis of the left proximal tibia. Tumor biopsy and immunohistochemistry confirmed the diagnosis of diffuse large B-cell lymphoma.

**Interventions:**

Both patients were treated with 6 courses of NHL-Berlin-Frankfurt-Münster-95.

**Outcomes:**

Both patients are in complete clinical remission with a follow-up of 27 and 18months after treatment, respectively.

**Lessons:**

PLB is a rare malignancy that is difficult to diagnose, particularly in children. Clinicians should increase the awareness of the disease and consider a differential diagnosis of bone lesions. Chemotherapy combined with radiotherapy is a favorable treatment for children with PLB. Early diagnosis and active treatment can improve patient prognosis.

## 1 Introduction

Primary lymphoma of the bones (PLB) is a rare malignancy, first described in adults in 1928 by Oberling and colleagues.^[1,2]^ PLB accounts for 3% to 7% of all primary bone malignancies, ∼5% of extranodal lymphomas, and less than 1% of all non-Hodgkin lymphoma (NHL) cases.^[1,3]^ The median age of PLB at diagnosis ranges from 45 and 60 years old.^[4]^ PLB is extremely uncommon in children, and only 2% to 9% of cases involve the bone as the primary site,^[5]^ with cases reported in literature sparse.^[6]^ Boys are diagnosed more frequently than girls, with a boy: girl ratio ranging from 1 to 1.8.^[1,2]^ PLB is mostly located in the femur or pelvis (50%) or the long bones of the upper limbs (20%), but can occur in other locations such as the ribs, mandible, or scapula (30%),^[7]^ with incidences in the tibia approaching 4.8% to 10%,^[8,9]^ PPB of the tibia is imitative and masquerades as chronic osteomyelitis, chondroblastoma, and Brodie abscess.^[10–12]^ PLB of the tibia has an excellent prognosis compared to bone NHL that presents in young patients at other sites.^[13]^ The clinical manifestations of PLB are nonspecific, with local pain the most common early symptom. This article reports 2 cases of primary lymphoma of the tibia in children to increase the awareness of the disease and to allow clinicians to consider a differential diagnosis of childhood bone lesions.

## 2 Case presentation

Informed written consent was obtained from the patients’ parents before publication of the case details and accompanying images.

### 2.1 Case 1

**Figure 1:**
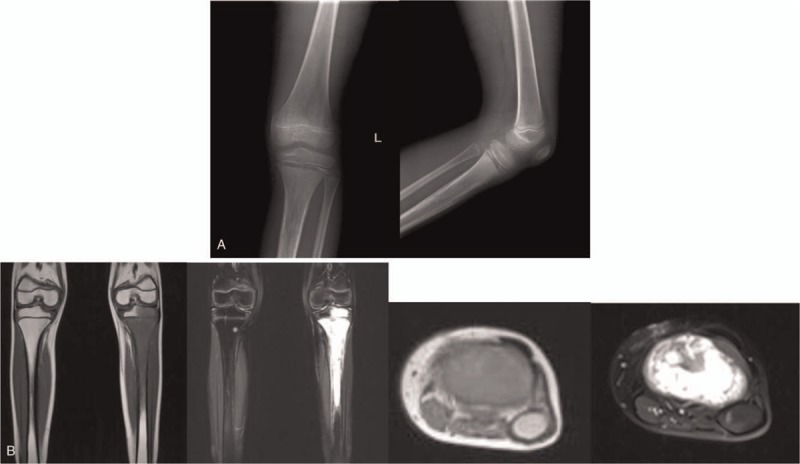
(A) The X-ray of knee showed bone mineral density reduction of left upper middle tibia, Local bone a periosteal reaction can be seen. (B) The MRI of knee showed an abnormal signal of the left upper middle tibia, metaphysis, and epiphysis. The lesion showed hypointensity on T1-weighted imaging and hyperintensity on T2-weighted imaging. Surrounding soft tissue was swelling with a 3 cm × 3 cm mass. MRI = magnetic resonance imaging.

A 9-year-old boy presented with swelling of the left calf and pain for 4 months after a fall. X-rays were normal and he was misdiagnosed with traumatic soft tissue injury. The pain increased 15 days before admission. Physical examination showed a 3 cm × 3 cm palpable mass in the left proximal calf with tenderness. Limited knee activity and a limited range of motion of the knee joints was observed at 20° to 60°. X-rays of the knee showed a loss of bone mineral density of the left upper middle tibia. Local bone periosteal reactions were also observed (Fig. 1A). Magnetic resonance imaging (MRI) revealed an abnormal signal of the left upper middle tibia, metaphysis, and epiphysis. The lesion showed hypointensity on T1-weighted imaging and hyperintensity on T2-weighted imaging. The surrounding soft tissue showed swelling with a 3 cm × 3 cm mass (Fig. 1B). Laboratory examinations revealed red blood cell (RBC) counts of 3.98 × 10^12^/L, white blood cell (WBC) counts of 8.5 × 10^9^/L, platelets (PLT) of 265 × 10^9^/L, alkaline phosphatase (ALP) of 151.2 U/L, lactate dehydrogenase (LDH) of 182 U/L, C-reactive protein (CRP) of 31.03 mg/L and erythrocyte sedimentation rates (ESR) of 19 mm/h. Grayish white tissue with focal hemorrhage was observed through surgical biopsy. Histological and immunohistochemistry confirmed a diagnosis of B-cell lymphoblastic lymphoma (B-LBL). Immunohistochemical staining revealed that CD10 (+), CD43 (+), PAX-5 (+), CD79a (+), CD99 (Scattered+), TdT (+), Ki-67(+60%), Vimentin (+), CD34 (−), CD7 (−), CgA (−), Syn (−), CD20 (−), CD3 (−), and MPO (−).

Positron emission tomography/computed tomography (PET/CT) revealed a hypermetabolic lesion in the left upper middle tibia, metaphysis, and epiphysis. Bone marrow examinations were normal. Patients were staged according to the International Pediatric NHL Staging System as stage IE disease.^[14]^ The patient was treated according to the NHL-Berlin-Frankfurt-Münster-95 (BFM-95) protocol.^[15]^ Complete clinical remission was achieved with a follow-up of 27 months.

### 2.2 Case 2

**Figure 2:**
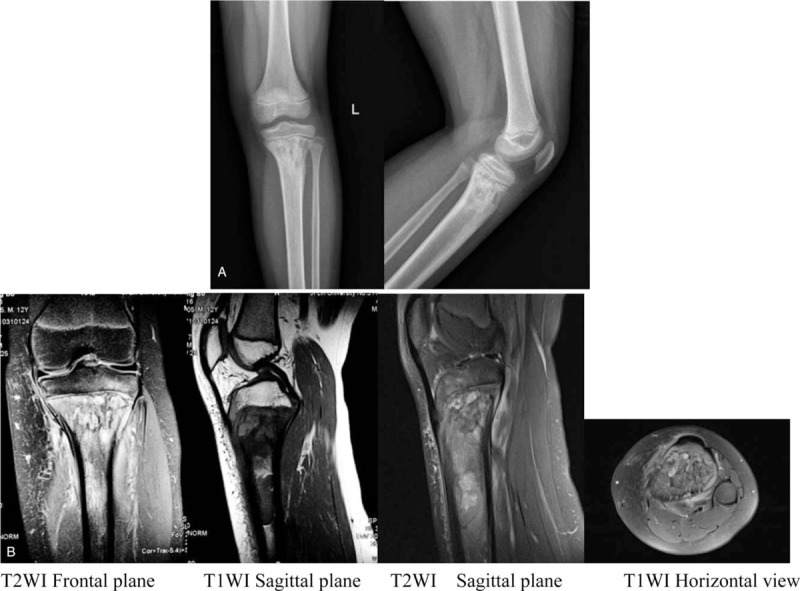
(A) The X-ray of knee showed a permeative lesion in the metaphysis and diaphysis of the left proximal tibia. (B) The MRI of knee demonstrated swelling of the left proximal tibia metaphysis and epiphysis and surrounding soft tissues. The lesion showed hypointensity on T1-weighted imaging, hyperintensity, and iso-intensity on T 2-weighted imaging. Local bone cortical discontinuity and a periosteal reaction can be seen. MRI = magnetic resonance imaging.

**Figure 3:**
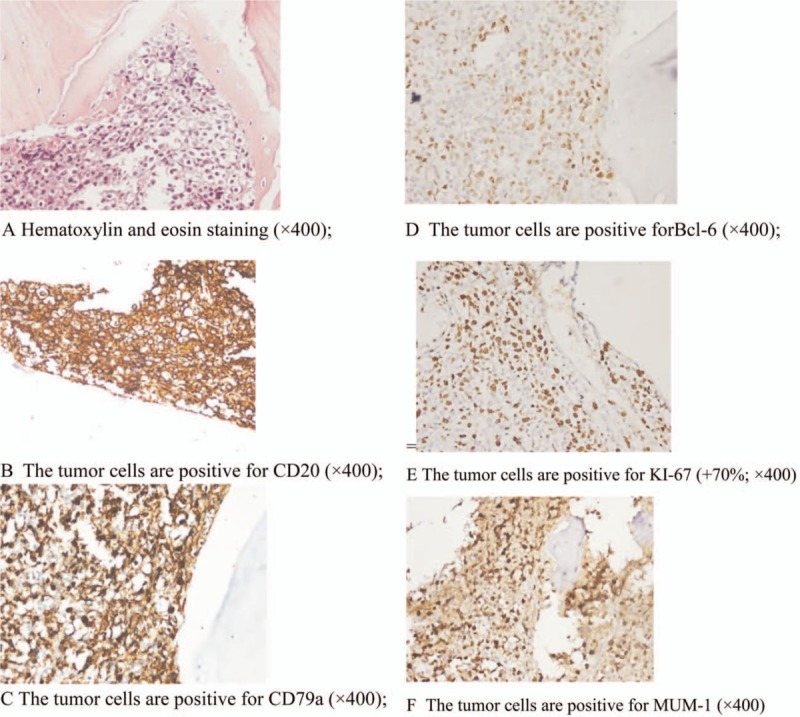
(A) Hematoxylin and eosin staining (×400). (B) The tumor cells are positive for CD20 (×400). (C) The tumor cells are positive for CD79a (×400). (D) The tumor cells are positive forBcl-6 (×400). (E) The tumor cells are positive for KI-67 (+70%; ×400). (F) The tumor cells are positive for MUM-1 (×400).

An 11-year-old boy presented with a 6-month history of intermittent left knee pain. Increased pain whilst walking was observed 10 days before admission. He was previously misdiagnosed with bone tuberculosis and chronic osteomyelitis following antibiotic therapy. His symptoms did not improve. There was no history of fever, chills, or night sweats. Physical examinations showed slight swelling of the left proximal tibia with mild tenderness. Local skin temperature and color were normal, and knee activity was unaffected. X-rays showed a permeative lesion in the metaphysis and diaphysis of the left proximal tibia (Fig. 2A). MRI demonstrated swelling of the left proximal tibia, metaphysis, and epiphysis of the surrounding soft tissue (Fig. 2B). The lesion showed hypointensity on T1-weighted imaging, hyperintensity, and iso-intensity on T2-weighted imaging. Local bone cortical discontinuity and periosteal reactions were observed. Laboratory examinations revealed an RBC count of 4.77 × 10^12^/L, a WBC count of 7.15 × 10^9^/L, a PLT count of 323 × 10^9^/L, an ALP of 278.3 U/L, a LDH of 252 U/L, a CRP of 3.03 mg/L, and an ESR of 34 mm/h. Tuberculosis related examinations were negative. Fish-like tumor tissue was noted from surgical biopsy of the left proximal tibia. The histological diagnosis was DLBCL. Immunohistochemical staining revealed (Fig. 3) CD20 (+), CD3 (scattered+), PAX-5 (+), CD79a (+), CD43 (partial+), Bcl-2 (−), CD10 (±), Bcl-6 (+), MUM-1 (+), c-Myc (+20%), and Ki-67(+70%).

**Figure 4:**
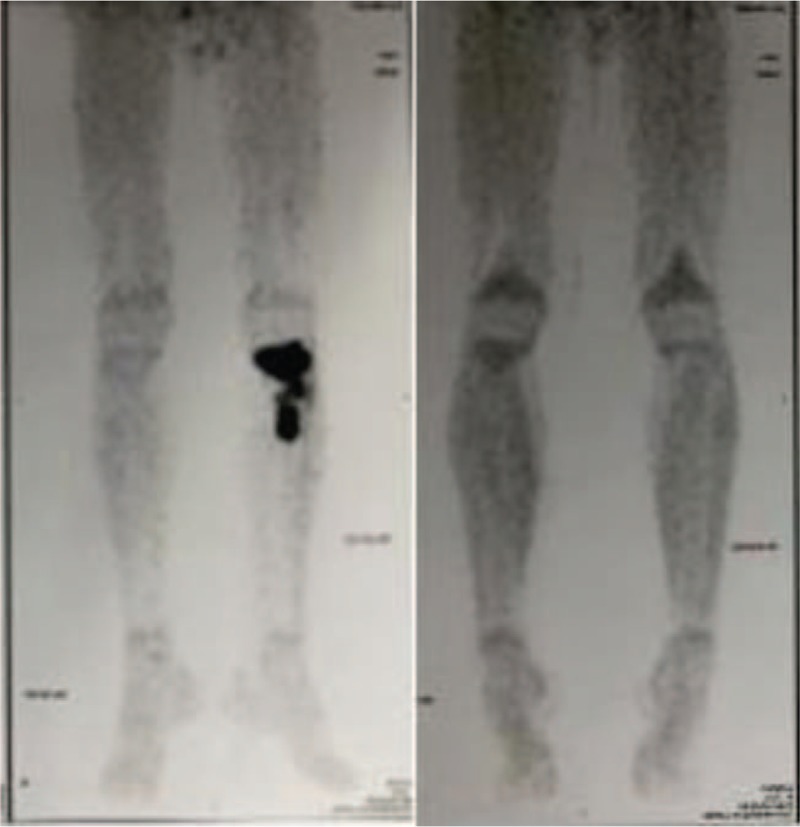
PET-CT before treatment (A) PET-CT after treatment (B). PET-CT = positron emission tomography/computed tomography.

PET/CT showed a hypermetabolic lesion of the left proximal tibia, SUV_Max_ 9.5, which was consistent with a malignant lesion (Fig. 4A). Bone marrow examinations were normal. The patient was staged according to the International Pediatric NHL Staging System as having stage IE disease. He was treated according to the NHL-BFM-95 protocol. Complete clinical remission was achieved with a follow-up of 18 months. PET/CT showed an inhibition of tumor activity following chemotherapy (Fig. 4B).

## 3 Discussion

PLB was defined in the 2013 World Health Organization classification of tumors of soft tissue and bone as a neoplasm composed of malignant lymphoid cells, producing 1 or more masses within the bone, in the absence of supraregional lymph node involvement or other extranodal lesions.^[3]^ The etiology of PLB remains uncertain^[16]^ but is related to risk factors including viral infections, immunodeficiency, genetic factors, osteomyelitis,^[4,10]^ trauma,^[17]^ total knee arthroplasty,^[18]^ exposure to noxious chemical agents, chemotherapy, and radiation.^[19]^ Case 1 had an established history of trauma.

Local pain is the most common initial symptom and the soft-tissue mass of the bone can be palpated.^[1,2]^ Pathologic fracture is an uncommon complication (≤10%).^[20]^ When the tumor occurs in the spinal column, different degrees of spinal cord compression occur.^[4]^ Some patients display anemia, osteolysis, and hypercalcemia-related symptoms.^[1,4]^ PLB patients rarely develop systemic or “B” symptoms such as fever, weight loss, and night sweats,^[1,3]^ as these would indicate systemic spread.^[1]^ Symptoms can persist for many months before seeking medical attention. Our patients both complained of local pain as the initial symptom and case 1 had a soft-tissue palpable mass around the knee.

All bones are potential sites for lymphoma development.^[4,16]^ In pediatric patients, the most common site is the femur,^[1,2]^ but disease can occur in the pelvis, spine, tibia, humerus, scapula, radius and talus, and maxilla.^[1,2,7,9,19,21]^ Up to 60% of cases involve a single bone.^[1]^ The metaphysis is the most common site of occurrence in long bones,^[22]^ whilst metaphysis and epiphysis involvement often reflect progressive disease.^[4]^ PLB is confined to the epiphysis in pediatric patients and is exceedingly rare.^[6]^ This site in our patients included the left tibia diaphysis, metaphysis, and epiphysis.

Radiographs tend to be normal,^[6,23]^ as observed in case 1. The main osteolytic manifestations include osteolytic bone destruction, cribriform bony absorption and on occasion, periosteal reactions in aggressive types,^[4,16]^ similar to that observed in case 2. CT often depicts a lytic lesion with cortical destruction.^[6]^ MRI typically exhibits low intensities on T1-weighted images and high intensities on T2-weighted images.^[1]^ X-rays, CT, and MRI often show bony abnormalities at tumor sites which can remain abnormal for years despite clinical remission, making it difficult to evaluate treatment responses.^[5,24]^ PET/CT has important clinical value for diagnosis, clinical staging, prognostic assessments, and post-treatment efficacy.^[25]^ This reflects the metabolic activity of malignant tumors and determines the extent of lymphoma involvement after the identification of distant metastases. In addition, PET/CT reduces radiation exposure after treatment and should be obtained 6 to 8 weeks after the completion of chemotherapy and 12 weeks after the completion of radiation therapy.^[26]^ However, mild metabolic activity on PET/CT scans fails to reliably differentiate the presence or absence of disease in patients with PLB that are in clinical remission. Marina et al^[5]^ suggested complementary roles of bone scintigraphy, MRI, and PET/CT in the diagnosis and treatment monitoring of children with PLB.

DLBCL is the most common subtype of PLB in children accounting for 70% to 80% of all PLB cases^[4]^ and including follicular lymphoma, peripheral T-cell lymphoma, marginal zone lymphoma and small lymphocytic lymphoma.^[1,2]^ Highly aggressive subtypes, such as Burkitt or lymphoblastic lymphoma are rare. Anaplastic large cell lymphoma is typically anaplastic lymphoma kinase-1 (ALK)-1 positive, has been reported.^[2,4]^ In this study, one of the cases were DLBCL, whilst the other was B-LBL.

On gross examination, PLB has a grayish-white meat-like appearance with focal hemorrhaging. Microscopically, the presence of diffuse and uniform lymphoma cells are observed. Tumor cells are large in size and possess consistent follicle centers or centroblastic cell types, often with nuclear cleavage.^[2,4]^ Unlike their adult counterparts, the pathology of large cell subtypes of pediatric DLBCL is poorly characterized, and typical centroblastic morphological features with nucleoli are rare. Multilobated nuclei are also uncommon.^[26]^ Primary B-cell lymphoma of the bones is typically positive for B-cell markers: CD20, CD21, CD97a, CD45,^[2]^ whilst immunoreactivity for CD75 and CD10 is variable.^[4]^ Primary T-cell lymphoma of the bone is often positive for T-cell markers: CD3, CD43, CD30.^[2,4]^ In cytogenetics and molecular studies, Huebner-Chan et al^[27]^ showed that the majority of PLB patients have IGH gene rearrangements, but lack BCL-2/JH gene rearrangements. Immune markers including BCL-2, P53, BCL-6, CD5, CD10, MUM-1, and ALK can determine pathological classification and treatment prognosis. However, unlike in adult PLB-DLBCL, CD10 negativity fails to predict poor prognosis.^[26]^

PLB can mimic infections, localized inflammation or other neoplastic changes.^[20]^ The differential diagnosis of PLB should include secondary lymphoma of the bone and other primary tumors of the skeletal system such as osteosarcoma, Ewing sarcoma, osteoid osteoma, fibrosarcoma, and malignant fibrous histiocytoma. Ewing sarcoma typically occurs in the epiphysis of children, with local pain as the main symptom, often accompanied by fever. Bone destruction during imaging formed from the sieve holes with onion skin-like periosteal reactions. WBCs, ESR and CRP increase upon laboratory examinations. In addition, non-neoplastic diseases such as osteomyelitis,^[10]^ eosinophilic granuloma,^[28]^ and bone tuberculosis should be considered for differential diagnosis. Acute osteomyelitis with severe systemic symptoms, and chronic osteomyelitis with bone sclerosis are both accompanied by soft tissue swelling, and PLB formation of the soft tissue mass. Eosinophilic granuloma is more common in children and occurs in bone sclerosis and periosteal reactions following long bone diaphysis. Bone tuberculosis often occurs in the epiphysis of the long shaft, often with a low fever, night sweats and other systemic symptoms. Tuberculosis of the knee joint leads to osteoporosis and abscess formation, and antituberculosis treatment is effective. Case 2 was previously misdiagnosed with bone tuberculosis and chronic osteomyelitis.

PLB is a rare but highly treatable tumor in children.^[26]^ Chemotherapy combined with radiotherapy represents a favorable treatment for PLB.^[1,7,29]^ Chemotherapy for the treatment of PLB in children is well established.^[1,30]^ Radiation therapy is sensitive to localized PLB,^[29]^ and is the treatment of choice for localized lymphoma of the bone.^[20]^ However, it has been reported that radiation therapy leads to muscle and bone damage at the radiation site and the occurrence of secondary malignant tumors.^[2]^ Studies^[1,30]^ have demonstrated that patients managed with chemotherapy alone have similar outcomes compared to those managed with concurrent radiation therapy. Radiation therapy is only suitable for children showing incomplete remission after chemotherapy or the progression of local lesions.^[30]^ Surgery is an unconventional treatment, but plays an important role in diagnosis and pathological fracture reduction and fixation, whilst minimizing potential delays in chemotherapy initiation.^[31]^

PLB in children is characterized by rapid progression, a higher incidence of micrometastasis, and a propensity for spread to the central nervous system, yet children display an improved prognosis.^[32]^ In a 5-year OS study, over 90% of patients were treated with standard chemotherapy.^[1,25,31]^ The prognosis is excellent when PLB is localized.^[29]^ Jamshidi and colleagues^[28]^ reported a combined modality therapy for stage IE PLB results with high survival rates, 5-year survival of 89% and a 5-year disease-free survival rate of 78%. However, the prognostic factors of PLB are not well-established.^[33]^ Prognosis is not influenced by bone involvement, the presence of soft tissue masses, or pathologic fractures.^[24,34]^ However, Subik and coworkes^[13]^ reported that proximal tibia lymphoma has an excellent prognosis, particularly in young patients. Children with PLB show more favorable outcomes than adults.^[24]^ Studies suggest that age is a prognostic factor for the survival of patients with PLB.^[1,2,7]^ Patients with PLB have survival rates of ∼90% compared to ∼62% in those aged over 60 years.^[34]^

## 4 Conclusions

PLB is a rare malignancy, particularly in children. The clinical presentation and radiological features of PLB are often non-specific, making clinical diagnosis difficult and easy to misdiagnose. Clinicians should increase their awareness of the disease and consider PLB as a differential diagnosis in children with bone lesions. Chemotherapy combined with radiotherapy is a favorable treatment option for children with PLB. Early diagnosis and active treatments can improve patient prognosis.

## Author contributions

SHQ drafted this manuscript. SHQ, LF and WZW analyzed and interpreted thepatient data. LHG evaluated the histopathological images and prepared the figures. XZ reviewed the clinical notes and edited the document. All authors read and approved the final manuscript.

**Data curation:** Haiqiang Suo.

**Investigation:** Li Fu and Zhiwei Wang.

**Resources:** Hanguang Liang, Zhe Xu.

**Supervision:** Wei Feng.

**Writing – original draft:** Haiqiang Suo.

**Writing – review & editing:** Wei Feng.
